# Comparison of the Ratio Between Anterior and Posterior Curvature Centered on the Thinnest Point (A/B Ratio) in Normal and Keratoconus Eyes

**DOI:** 10.1167/tvst.15.6.34

**Published:** 2026-06-26

**Authors:** Eszter Szalai, Renato Ambrosio, Elias Flockerzi, Berthold Seitz, Hamed Momeni-Moghaddam, Mohammad-Reza Sedaghat, Michael W. Belin

**Affiliations:** 1 Department of Ophthalmology, University of Pecs Medical School, Pecs, Hungary; 2 Federal University of the State of Rio de Janeiro, Ophthalmology Department, Rio de Janeiro, Brazil; 3 Rio de Janeiro Corneal Tomography and Biomechanics Study Group, Rio de Janeiro, Brazil; 4 Department of Ophthalmology, Saarland University Medical Center, Homburg, Germany; 5 Rehabilitation Sciences Research Center, Zahedan University of Medical Sciences, Zahedan, Iran; 6 Eye Research Center, Mashhad University of Medical Sciences, Mashhad, Iran; 7 University of Arizona, Department of Ophthalmology & Vision Sciences, Tucson, AZ, USA

**Keywords:** keratoconus, corneal tomography, corneal curvature, radius of curvature

## Abstract

**Purpose:**

The purpose of this study was to evaluate in a large multi-national database (3873 eyes) the anterior to posterior radius of curvature ratio taken from a 3.0 mm optical zone centered on the thinnest point (A/B ratio) and determine its clinical applicability in discriminating healthy from ectatic eyes.

**Methods:**

Databases from three keratoconus centers (Homburg, Germany, Mashhad, Iran, and Rio de Janeiro, Brazil) were analyzed. Databases included healthy (*n* = 2061) and keratoconus eyes (*n* = 1812). Anterior and posterior radius of curvature values were determined by the “A” and “B” parameters from the Belin ABCD staging. The ratio was calculated by dividing the “A”/“B” parameter in radius of curvature.

**Results:**

Diagnostic accuracy expressed by area under the curve (AUC) was 0.951. Threshold value for classifying individuals as positive or negative was >1.27 with sensitivity of 85.8% and specificity of 95.2%.

**Conclusions:**

The A/B ratio centered on the corneal thinnest point shows high specificity, and moderate sensitivity. In the database of nearly 4000 eyes only 2 healthy eyes had an A/B ratio greater than 1.300, and no healthy eyes were above 1.335. An A/B ratio greater than 1.300 was highly suggestive of variance from normal with a specificity of 99.9%. Our results suggest that the A/B ratio may be a useful clinical tool in determining variance from normal.

**Translational Relevance:**

The A/B ratio centered on the thinnest point offers a simple, clinically applicable parameter with high specificity for detecting deviations from healthy corneal morphology. Its use may enhance early identification of ectatic disease and support more precise screening in refractive surgery candidates.

## Introduction

Prior to the advent of modern imaging, keratoconus was diagnosed by clinical signs and symptoms. High degrees of astigmatism, Munson's sign, Rizutti's sign, the presence of a Fleischer ring, distorted keratometry mires, and decreased visual acuity were the hallmarks of disease.^1^ The advent of computerized (Placido) videokeratoscopy by Klyce in 1984 led to additional parameters derived from the anterior corneal surface.^2^ Asymmetric bow-tie curvature patterns, inferior-superior power asymmetry (I-S index), and the KISA index were just a few.^3^^,^^4^ Anterior segment tomography added the ability to measure the posterior corneal surface in addition to the anterior surface and to generate a full corneal thickness map.^5^ This added information allowed for an earlier identification of ectatic disease.^6^

Keratoconus is typically described as a condition where the cornea thins and bulges leading to a steepening of the anterior surface (i.e. in more advanced cases). Corneal thinning is usually maximal at the cone, whereas the corneal periphery approaches normal thickness values. The point of maximal thinning has been shown to have the highest correlation to the cone apex and is typically displaced in the inferior-temporal location.^7^^,^^8^ The difference in apical thickness reading compared to minimal corneal thickness has been reported as high as 93 microns in keratoconic corneas.^9^ This change in corneal thinning, from minimal values at the cone to a more normal periphery, led to the development by Ambrosio in 2006 of pachymetric progression graphs and indices.^10^^,^^11^ Any change in corneal thickness in which the relative thickness differs from normal would require that the relationship between the anterior and posterior corneal curvature also change. The anterior to posterior corneal ratio in healthy eyes is in the range of 1.21 ± 0.02 when centered at the corneal apex.^12^ Anterior corneal steepening associated with corneal thinning would mandate a greater change in the posterior cornea relative to the anterior corneal surface. Increased posterior elevation and a steeper posterior best-fit-sphere relative to the anterior surface have been previously observed.^13^^–^^16^

Prior studies comparing the anterior and posterior curvature ratio (AP ratio) evaluated the radius of curvature from multiple optical zones centered on the cornea apex and found this ratio to have poor predictive ability. These prior studies involved relatively few subjects and measured curvature only centered on the corneal apex.^17^^,^^18^ In ectatic disease, the thinnest point more closely approximates the cone, and readings can vary significantly from the reading at the corneal apex.^9^^,^^18^^,^^19^ This study evaluated whether the anterior to posterior curvature ratio measurements taken at the thinnest point, as opposed to the corneal apex, would have clinically useful discriminatory potential.

This retrospective study evaluates in a combined large multi-national database (3873 eyes) the relationship between the anterior corneal curvature to the posterior curvature (A/B ratio) taken from a 3-mm zone centered on the corneal thinnest point (“A” and “B” parameter from the ABCD keratoconus classification)^20^ and to determine whether this ratio, centered on the thinnest point, has sufficient discriminatory ability to separate healthy and keratoconus eyes.

## Methods

In this retrospective study, established tomographic databases (Pentacam HR, OCULUS GmbH, Wetzlar, Germany) from three keratoconus centers (Homburg Keratoconus Center, Saarland University Medical Center, Homburg, Germany,^21^^–^^23^ Didar Eye Clinic, Mashhad, Iran,^24^^–^^26^ and Instituto de Olhos Renato Ambrósio and Rio de Janeiro Corneal Tomography and Biomechanics Study Group, Rio de Janeiro, Brazil)^27^^,^^28^ were combined for analysis. All three databases included both healthy (*n* = 2061) and keratoconus eyes (*n* = 1812) and were previously published and peer reviewed (Table 1).^21^^–^^28^ This extensive database includes all degrees of disease severity from very mild to severe disease. When both eyes were available, the eye with the lower Topographic Keratoconus Classification (TKC) value was chosen. There were 20.2% of the keratoconic database that had a Pentacam TKC score below 1.0 (OCULUS GmbH, Wetzlar, Germany), with K values as low as 36.3 diopters signifying very early and/or subclinical disease. The Pentacam TKC score is a composite of a number of anterior surface parameters (i.e. topographically derived) but does not incorporate data from the posterior cornea. All subjects underwent complete eye examinations minimally including manifest refraction, slit lamp examination, and anterior segment tomography.

**Table 1.** Number of Healthy Subjects and Patients With Keratoconus

Center	Healthy Subjects	Keratoconus Subjects
Instituto de Olhos Renato Ambrósio and Rio de Janeiro Corneal Tomography and Biomechanics Study Group, Rio de Janeiro, Brazil	1681	1183
Homburg Keratoconus Center (HKC), Saarland University Medical Center, Homburg, Germany	109	381
Didar Eye Clinic, Mashad, Iran	271	248
Total	2061	1812

Anterior radius of curvature (ARC) and posterior radius of curvature (PRC) measurements were determined by the “A” and “B” parameters from the Belin ABCD staging.^20^ Each measurement is a global reading taken from a 3.0 mm optical zone centered on the thinnest point of the cornea. The anterior to posterior ratio (A/B) was then calculated (ARC/PRC).

### Statistical Analysis

Data were analyzed using the MedCalc version 14.8.1. For each data set, mean, standard deviation (SD), and 95% confidence interval (95% CI) for the mean were calculated. The Kruskal-Wallis test and Mann-Whitney *U* test were carried out for comparison between groups or variables. A receiver operating characteristic (ROC) curve was created, which plots the rate of true positives (true positive rate = sensitivity) against the rate of false positives (1 – true negative rate = specificity).^29^ ROC analysis provided appropriate cutoff points (threshold values) for the measured parameters. Sensitivity, specificity, and positive and negative predictive values for each cutoff point were also calculated. The accuracy of the screening tests was measured by the area under the ROC curve (AUROC). A *P* value below 0.05 was considered statistically significant. The best cutoff values for the A/B ratio were determined by identifying the point on the ROC curve that maximized the Youden index.

## Results

The mean age of the healthy subjects and the patients with keratoconus were 34.3 ± 13. 0 years (ranging from 7 to 90 years) and 32.8 ± 11.2 years (ranging from 7 to 80 years), respectively (*P* = 0.053). The ARC, PRC, and A/B ratio measurements in the 3-mm optical zone centered on the thinnest point of the cornea are summarized in Table 2. In all three parameters, statistically significant differences were obtained between the healthy and keratoconus groups (*P* < 0.0001, Mann-Whitney *U* test).

**Table 2.** Anterior (ARC), Posterior Radius of Curvature (PRC), and Anterior to Posterior Radius of Curvature Ratio (A/B Ratio) Measurements in the 3 mm Optical Zone Centered on the Thinnest Point of the Cornea

	Subjects^b^		
	Healthy	Keratoconus	*P* Value^a^
ARC	7.770 ± 0.253 (7.759–7.781)	6.788 ± 6.397 (6.579–6.817)	<0.0001
PRC	6.338 ± 0.240 (6.328–6.348)	5.135 ± 0.631 (5.106–5.164)	<0.0001
A/B ratio	1.226 ± 0.023 (1.225–1.227)	1.328 ± 0.065 (1.325–1.331)	<0.0001

a Mann-Whitney U test.

b Mean ± standard deviation (95% confidence interval).

**Figure 1.**
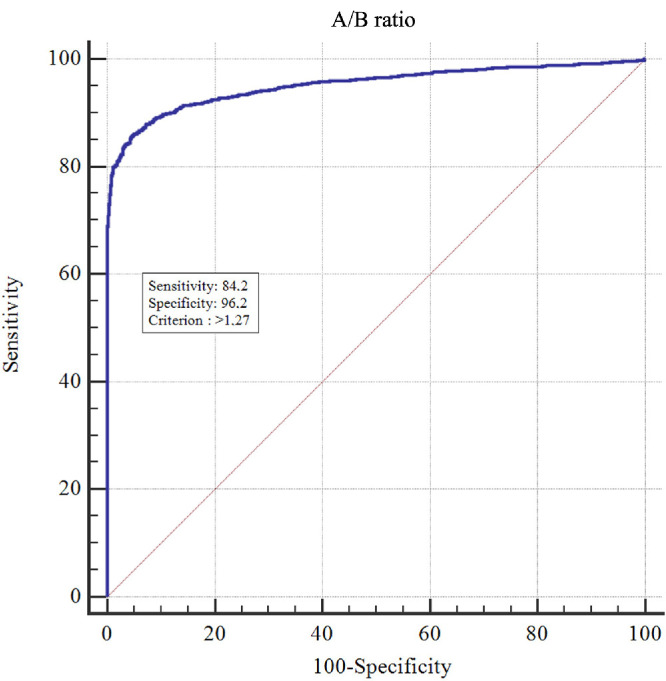
Receiver operating characteristic curve for anterior to posterior radius of curvature ratio (A/B ratio) showing the A/B ratio cutoff point (1.27) with the best separation (minimal false negative and false positive results) between the two groups.

**Figure 2.**
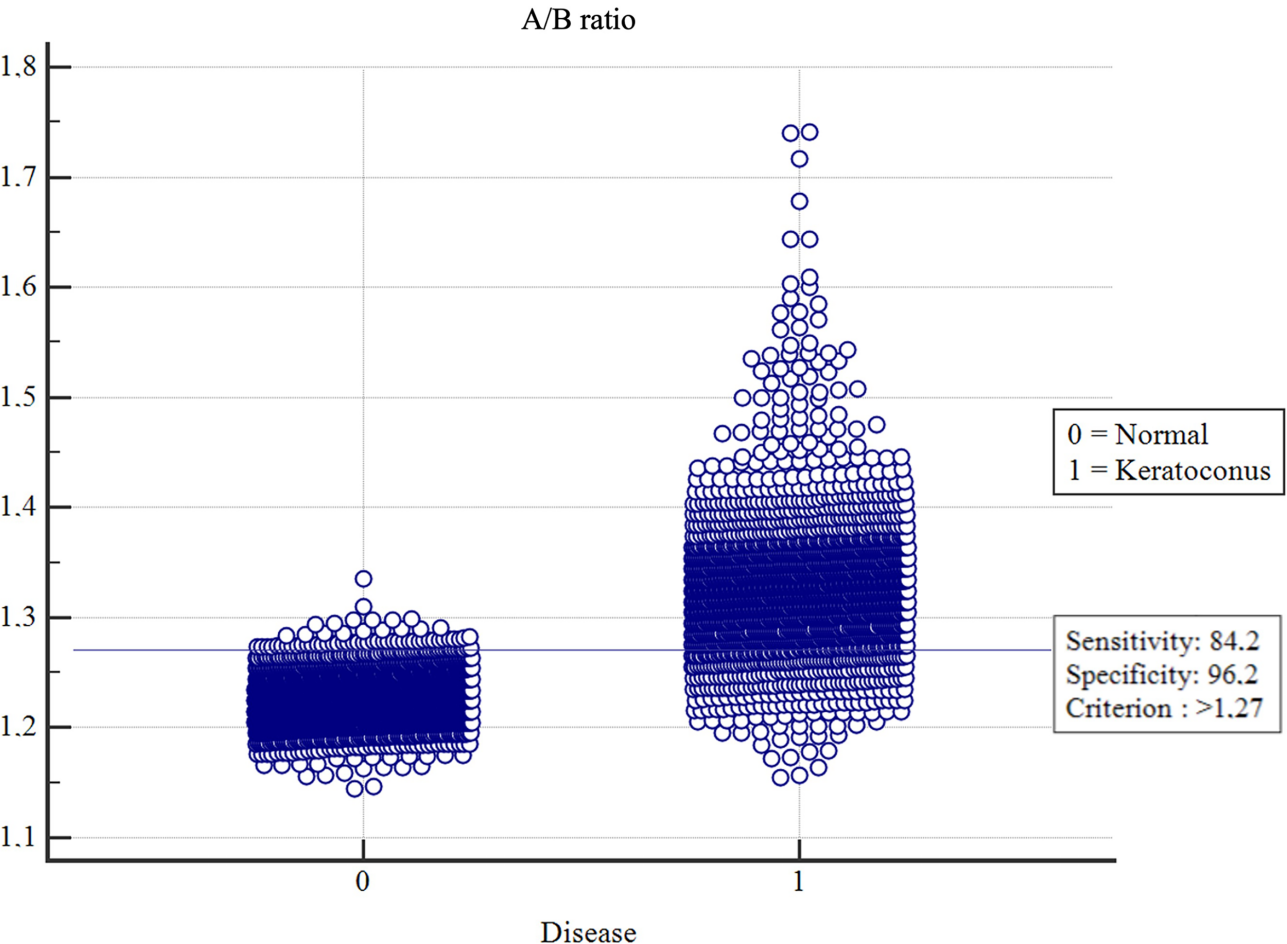
Interactive dot diagram (scattergram) for anterior to posterior radius of curvature ratio (A/B ratio). The graph shows the A/B ratio cutoff point (1.27) with the best separation (minimal false negative and false positive results) between two groups. The scattergram shows the very tight grouping of the healthy population and the wider dispersal for the keratoconus population.

The ROC curve and scattergram plot showing the A/B ratio measurement overlap between the healthy and keratoconus groups are displayed in Figures 1 and 2. The diagnostic accuracy for A/B ratio, expressed by the AUROC values, was 0.951 (standard error = 0.004, 95% CI = 0.944–0.958). A best cutoff value (corresponds to the threshold value that maximizes the Youden index) for classifying individuals as positive (keratoconus) or negative (healthy) was >1.27 for A/B ratio with a sensitivity of 85.8%, a specificity of 95.2%. The likelihood ratios for different A/B intervals are shown in Table 3. Only two healthy corneas (2/2061) had an A/B ratio greater than 1.300 with a sensitivity of 61.04% (95% CI = 58.70–63.30), a specificity of 99.90% (95% CI = 99.60–100), and no healthy eyes were above 1.335 with a sensitivity of 40.51% (95% CI = 38.20–42.80), or a specificity of 100% (95% CI = 99.80–100) in the healthy group.

**Table 3.** Anterior to Posterior Radius of Curvature Ratio (A/B Ratio) Interval Likelihood Ratios

A/B Ratio Interval	Positive, Keratoconus	Negative, Healthy	Likelihood Ratio (95% CI)
1.1–1.2	15	230	0.074
			(0.044–0.125)
1.2–1.3	564	1829	0.351
			(0.327–0.376)
1.3–1.4	1047	2	595.438
			(148.931–2380.612)
1.4–1.5	149	0	∞
			(21.117–∞)
1.5–1.6	29	0	∞
			(4.032–∞)
1.6–1.7	5	0	∞
			(0.622–∞)
1.7–1.8	3	0	∞
			(0.342–∞)
Total	1812	2061	

To evaluate the robustness of the A/B ratio across our different geographic populations, we performed separate ROC analyses. All three populations demonstrated strong diagnostic performance with similar area under the curve (AUC) values and diagnostic characteristics. The ROC curves showed a high degree of overlap, indicating consistent discriminatory ability across different geographic settings.

We performed additional ROC analysis specifically for early keratoconus, defined as TKC ≤1. Eyes with TKC ≤1 were compared with healthy controls. The A/B ratio showed strong discriminatory ability for early keratoconus, with an AUC of 0.875 (95% CI = 0.816–0.927). Using the Youden-derived cutoff of 1.257, sensitivity was 68.2% and specificity was 92.0%. These results suggest that the A/B ratio retains clinically meaningful diagnostic performance even in early-stage keratoconus.

## Discussion

Keratoconus is a corneal ectatic disease with high degrees of intrasubject asymmetry.^30^ The location of the cone (area of maximal corneal steepness) and the corneal thinnest point typically are coincident or very close to each other.^8^ Historically, measurements were typically taken at the corneal apex. Measurements over the cone, as opposed to the corneal apex, have advantages in that they better represent disease severity. This was historically known and led to the development of a modification to the standard keratometer, which allowed for a moveable fixation light allowing for off-axis readings.^31^^,^^32^ Computerized videokeratoscopes, however, rely more on central apical readings and the standard axial curvature maps often misrepresents the true cone location.^8^^,^^33^ Tomography (either Scheimpflug or optical coherence tomography [OCT]) does not rely upon corneal reflection and as such allows for off-axis shape analysis. Accurate cone location is important in selective cross-linking, photo-therapeutic keratectomy (PTK) for keratoconus aberration reduction, and intracorneal ring placement.^24^^,^^34^^,^^35^ Curvature analysis centered on the corneal thinnest point is also utilized in the Belin ABCD keratoconus classification.^20^

Our study looked at the potential of a simple single parameter (A/B ratio) to differentiate healthy corneas from corneas with keratoconus and to determine whether analysis at the thinnest point has sufficient discriminatory potential to be clinical useful. An AUROC value of 0.951 shows that the A/B ratio is very powerful in discriminating patients with keratoconus from healthy individuals. As a single identifying parameter, the A/B ratio shows high specificity (95.2%). In our combined database of nearly 4000 eyes only 2 healthy eyes had an A/B ratio greater than 1.300, and no healthy eyes were above 1.335 (see Table 3). An A/B ratio, when centered on the thinnest point, greater than 1.300 was therefore highly suggestive of ectatic disease with a specificity of 99.9% (excluding other prior pathology). Although the healthy population's A/B ratio was tightly grouped, the keratoconus population showed significant scatter (see Fig. 2). The A/B ratio was significantly less sensitive (85.8%) in detecting ectatic disease, so whereas the A/B ratio can indicate variance from healthy to a very high level, its ability to detect all degrees of keratoconus is more limited. However, A/B values in excess of 1.300 should be viewed as highly suspicious.

This study represents the largest number of subjects evaluated (3873) and used a previously documented, multi-national database of both healthy and keratoconus subjects representing patients from Europe, the Middle East, and South America. Although the keratoconic database consisted of a wide range of disease severity, patients with highly asymmetric, but healthy tomography, and cases with suspicious biomechanical parameters were not evaluated. Whereas there are some clinical limitations to the application of the A/B ratio as a single screening parameter (and is not being proposed as such) our study does support the concept that corneal shape analysis based on the thinnest point has inherent advantages over apical based analysis. Compared with earlier published studies solely evaluating topographic (anterior corneal) parameters, cutoff values for specificity for CKI ranged from 0.686 to 0.982, for K1 0.657 to 0.982, for IHA 0.794 to 0.956, IHD 0.457 to 0.988.^36^ Direct comparisons to prior published studies are difficult as the subject pool varies as do the definitions of what constitutes healthy and keratoconic corneas. This very large variance is likely due to both different patient databases and different definitions of disease making direct comparisons to our results problematic.

The goal of this study was to determine the performance and potential utility of a single simple parameter and to determine whether centering measurements on the corneal thinnest point has diagnostic advantages over apex-based readings. Whereas the performance of the A/B ratio exceeds those of prior studies, which were centered on the cornea apex, a direct comparison using the current database was not possible. A direct comparison using corneal versus cone apex readings with the study database, while optimal, was not possible due to (1) the retrospective nature of the study, and (2) the export function of the Pentacam includes the “A” and “B” parameters as these are available Pentacam values in the Belin ABCD classification and Belin ABCD progression display, but comparable values over the corneal apex are not available. Although the A/B ratio performance is respectable, it falls below prior AUROC values for more complex multiple combined tomographic parameters utilized in a regression analysis, such as the final “D” in the Belin/Ambrosio display.^37^^,^^38^
